# Draft Genome Sequences of Two Novel Amoeba-Resistant Intranuclear Bacteria, “*Candidatus* Berkiella cookevillensis” and “*Candidatus* Berkiella aquae”

**DOI:** 10.1128/genomeA.01732-15

**Published:** 2016-02-18

**Authors:** Yohannes T. Mehari, Brock A. Arivett, Anthony L. Farone, John H. Gunderson, Mary B. Farone

**Affiliations:** a Department of Biology, Middle Tennessee State University, Murfreesboro, Tennessee, USA; b Department of Biology, Tennessee Technological University, Cookeville, Tennessee, USA

## Abstract

“*Candidatus* Berkiella cookevillensis” and “*Candidatus* Berkiella aquae” are obligate intranuclear endosymbionts of freshwater amoebae. Here, we present the draft genome sequences of these two bacteria, with total sizes of 2,990,361 bp and 3,626,027 bp, respectively.

## GENOME ANNOUNCEMENT

Two novel bacteria were isolated from freshwater amoebae found in aquatic biofilms in Cookeville, TN, USA. “*Candidatus* Berkiella cookevillensis” strain CC99 was recovered from an infected amoeba from a cooling tower, and “*Candidatus* Berkiella aquae” strain HT99 was collected from an infected amoeba in an outdoor hot tub. The 16S rRNA gene sequences were 100% (CC99) and 96% (HT99) similar to sequences from uncultured organisms in the NCBI GenBank database. CC99 had 100% identity to an uncultured isolate from a South Korean oyster shell waste pile. Preliminary analyses showed 94% 16S rRNA sequence similarity between the two isolates and <92% similarity to *Legionella* or *Coxiella* species (1). Similar bacteria resembling *Legionella* spp. have been recovered from free-living amoebae, some of which are associated with respiratory disease (2). However, unlike *Legionella* spp., both bacteria invade and replicate in the host nucleus, resulting in complete lysis of the host cells within 2 to 4 days.

The bacteria were maintained in the laboratory by continuous passage in *Acanthamoeba polyphaga*. For genome sequencing, the bacteria were purified from infected amoebae by Renografin density gradient centrifugation, as described by Shannon and Heinzen (3). Total DNA was extracted using the DNeasy blood and tissue kit (Qiagen, Valencia, CA). Whole-genome sequencing was performed using a 250-bp paired-end MiSeq platform (Illumina, San Diego, CA) at the Institute for Genome Sciences (University of Maryland School of Medicine, Baltimore, MD). A total of 11,839,536 and 11,009,736 reads were generated for CC99 and HT99, respectively. The reads were subsampled to genome coverages of between 60 and 200× in 10× increments and assembled *de novo* with MaSuRCA (4). For “*Ca.* Berkiella cookevillensis” strain CC99, 44 contigs were generated, for a combined draft genome size of 2,990,285 bp, with a G+C content of 37.9%. The draft genome of “*Ca.* Berkiella aquae” strain HT99 contains 56 contigs, with a total length of 3,625,323 bp and a G+C content of 39.5%.

Prokka and the IGS Annotation Engine were used for structural and functional annotation of the sequences (5, 6), and Manatee was used to view and curate the annotations (http://manatee.sourceforge.net/). For isolate CC99, 2,598 genes were predicted, with 2,557 predicted coding sequences (CDSs). For HT99, 3,231 genes and 3,189 CDS regions were identified. Using RNAmmer and tRNAscan-SE, the CC99 genome is predicted to contain 3 rRNA and 40 tRNA genes, while the HT99 genome is predicted to contain 3 rRNA and 41 tRNA genes.

Also noteworthy is the existence of several of the *dot* or *icm*-type IV secretion system (T4SS) genes in both isolates, which is important for the pathogenesis of both *Legionella pneumophila* and *Coxiella burnetii*, bacteria similar to CC99 and HT99 (7, 8). The new draft genome sequences from both “*Ca.* Berkiella cookevillensis” and “*Ca.* Berkiella aquae” will allow for more comprehensive phylogenetic analyses of these novel bacteria and greater understanding of their intranuclear invasion and growth. The intranuclear lifestyles of the bacteria in amoebae might also enhance horizontal gene transfer between organisms, consistent with the idea of amoebae as evolutionary “melting pots” (9).

### Nucleotide sequence accession numbers.

These whole-genome shotgun projects, associated with BioProject PRJNA289553 (“Novel amoeba-resistant bacteria”), have been deposited at DDBJ/EMBL/GenBank as Berkiella cookevillensis CC99 under the accession no. LKHV00000000 and Berkiella aquae HT99 under the accession no. LKAJ00000000. The versions described in this paper for “*Ca.* Berkiella cookevillensis” strain CC99 and “*Ca.* Berkiella aquae” strain HT99 are versions LKHV01000000 and LKAJ01000000, respectively.
